# Numerical study of the effects of minor structures and mean velocity fields in the cerebrospinal fluid flow

**DOI:** 10.1186/s12987-024-00604-x

**Published:** 2024-12-18

**Authors:** Ziyu Wang, Mohammad Majidi, Chenji Li, Arezoo Ardekani

**Affiliations:** School of Mechanical Engineering, Purdue University, 585 Purdue Mall, West Lafayette, 47907 IN USA

**Keywords:** Cerebrospinal fluid, Intrathecal injection, Computational fluid dynamics, Lagrangian velocity

## Abstract

The importance of optimizing intrathecal drug delivery is highlighted by its potential to improve patient health outcomes. Findings from previous computational studies, based on an individual or a small group, may not be applicable to the wider population due to substantial geometric variability. Our study aims to circumvent this problem by evaluating an individual’s cycle-averaged Lagrangian velocity field based on the geometry of their spinal subarachnoid space. It has been shown by Lawrence et al. (J Fluid Mech 861:679–720, 2019) that dominant physical mechanisms, such as steady streaming and Stokes drift, are key to facilitating mass transport within the spinal canal. In this study, we computationally modeled pulsatile cerebrospinal fluid flow fields and Lagrangian velocity field within the spinal subarachnoid space. Our findings highlight the essential role of minor structures, such as nerve roots, denticulate ligaments, and the wavy arachnoid membrane, in modulating flow and transport dynamics within the spinal subarachnoid space. We found that these structures can enhance fluid transport. We also emphasized the need for particle tracking in computational studies of mass transport within the spinal subarachnoid space. Our research illuminates the relationship between the geometry of the spinal canal and transport dynamics, characterized by a large upward cycle-averaged Lagrangian velocity zone in the wider region of the geometry, as opposed to a downward zone in the narrower region and areas close to the wall. This highlights the potential for optimizing intrathecal injection protocols by harnessing natural flow dynamics within the spinal canal.

## Introduction

Within the human spinal canal, the cerebrospinal fluid (CSF) plays a crucial role in transporting dissolved nutrients and waste products throughout the subarachnoid space (SAS) because of its pulsatile nature. CSF flow is driven by the periodic blood volume changes subject to the conservation of mass (i.e., Monro-Kellie doctrine) [2]. Intrathecal (IT) injection leverages this fluid dynamic property of the CSF to deliver therapeutic molecules to the central nervous system (CNS), making it an efficient CNS-targeted drug delivery method.

The study of optimizing IT drug delivery garners considerable interest due to its crucial role in enhancing medical treatments and patient health outcomes. Many researchers have developed computational fluid dynamics (CFD) models to investigate drug transport within the spinal SAS [1, 3–11]. Some of these studies have evaluated the influence of various injection parameters, such as the injection rate, volume, angle and location, in an attempt to optimize IT injection protocols [8–11]. Di Chiro (1964) detected the tracers administered in the lumbar area in the basal cisterns after one hour [12]. This observation shows that a slow net movement of CSF exists, with a velocity of approximately 0.1 mm/s which is much smaller than the pulsatile velocities on the order of a few cm/s during cardiac and respiratory cycles [12, 13]. Consequently, computational efforts become considerably demanding to track the intrathecally injected drugs till they leave the spinal SAS, especially when the number of computational cells needs to be amplified to resolve small structures. The extensive computational requirements present a significant challenge for the comprehensive exploration of the parameter space. Additionally, due to resource constraints, some studies resorted to neglecting small structures, and most studies rely on geometries derived from a limited number of subjects (<3). This can limit the scope and applicability of the findings because of the high degree of inter-individual and position-induced geometric variability that characterizes the spinal canal [7, 14, 15] and associated variability of CSF flow fields within the spinal SAS [7, 14, 16, 17]. Distinct drug transport patterns have been observed across various geometries, each derived from patient-specific medical imaging, following drug administered intrathecally under identical injection parameters [10]. This geometric variability exists not only in individuals with diseases or injuries that cause changes in the geometry of the spinal SAS but also among healthy subjects. This could be attributed to the contradictions in the key findings of the previous studies, for example, the impact of the bolus rate [8, 10, 11, 18].

If the anatomical position remains unchanged, each individual’s CSF flow field will stay nearly periodic over the long term, and the cycle-averaged Lagrangian velocity field will be approximately unchanged over time. Therefore, the process of optimizing injection protocols is essentially finding the optimal initial drug distribution when the dynamics of the injection jet have dissipated, and the baseline pulsatile CSF flow regains its dominance over the flow field. To deliver drugs in the most efficient and effective way, the injection protocols should be designed to achieve the best possible initial drug distribution tailored to the individual’s specific cycle-averaged Lagrangian velocity field in spinal SAS. As such, it is important to establish a universal principle to evaluate the cycle-averaged Lagrangian velocity field for an individual, using the geometry of their spinal SAS as a basis. Employing such a universal principle can simplify the injection parametric study: instead of simulating both the injection and drug transport from the injection site to the targeted area, the focus can be narrowed down to simulating merely for initial drug distribution upon the injection.

In order to formulate such a universal principle, it is essential to uncover the relationship between the geometry of the spinal canal and the cycle-averaged Lagrangian velocity field. This can be accomplished by investigating the dominant physical mechanisms within the spinal canal that facilitate the observed tracer transport at a speed approximating 0.1 mm/s [12]. The hypothesized mechanisms that could play a part in this transport process include shear-enhanced diffusion, also known as Taylor dispersion [19], steady streaming, as well as Stokes drift or eddy-induced movements. The shear-enhanced diffusion arises from the coupling of transverse diffusion with the radial shear and azimuthal shear inherent to the longitudinal pulsatile flow through the spinal SAS. However, suppose CSF oscillatory flows through a circular pipe possessing a radius equivalent to that of the spinal canal, the produced effective diffusivity is only approximately ten times greater than the molecular diffusivity, *D* [20]. This results in a negligible effective diffusion velocity on the order of $2 \times 10^{-6}$ mm/s, if $D = 10^{-10}$ $\mathrm {m^2/s}$. Furthermore, Lawrence et al. (2019) [1] provided theoretical evidence asserting that Taylor dispersion becomes negligible when considering pulsatile CSF flow within the spinal SAS. An alternate mechanism, steady streaming (SS), is suggested to account for the bulk motion of tracers observed in the spinal SAS by producing a nonzero nonuniform cycle-averaged Eulerian velocity field [21]. It can arise from oscillatory flow with zero temporal mean and has been previously demonstrated to be a significant factor in various biological oscillatory flows, such as respiratory and cardiovascular flows [22–24]. In addition, the Stokes drift (SD), generated by the gradient of the longitudinal pulsation amplitude in the transverse direction, can lead to a discrepancy between the Eulerian and tracer (i.e., Lagrangian) velocities [25].

A correlation has been observed between the geometry of spinal SAS and the patterns of steady streaming by Coenen et al. (2019) [7]: upward cycle-averaged Eulerian velocity field is found on the wider side of the spinal canal, while downward field is seen on the narrower side. Additionally, there’s a net azimuthal motion moving from the wide side to the narrow side, and the magnitude of steady streaming diminishes as the canal eccentricity decreases. These findings suggest that establishing a universal principle could be a feasible approach. However, the patterns of steady streaming in the presence of minor structures within the spinal canal, such as nerve roots, denticulate ligaments, and the wavy arachnoid membrane, remain to be explored, given that several studies have suggested their potential to alter the flow field [3, 8, 10, 11, 26, 27]. Furthermore, it is necessary to compute and scrutinize the Stokes drift velocity, building on the seminal analysis by Lawrence et al. (2019) [1], which has addressed its impact on the Lagrangian velocity.

The primary objective of our research is to identify a universal principle that will guide the customization of IT injection protocols for each individual patient. In the present study, the pulsatile CSF flow fields and Lagrangian velocity fields within canonical spinal SAS geometries were numerically computed. This study presents the cycle-averaged Eulerian, Stokes drift, and Lagrangian velocity fields of the pulsatile CSF flow within the representative vertebral column. The influence of minor anatomical structures on drug delivery was also analyzed. Moreover, the contributions made by the major mechanisms: steady streaming and Stokes drift to the mass transport, were examined, respectively.

## Methods

In this study, the pimpleFoam solver in OpenFOAM was utilized to handle unsteady, incompressible flow. We utilized the Finite Volume Method with an Euler implicit time scheme, ensuring first-order accuracy in time, and a second-order accurate discretization scheme in space. The numerical simulations were conducted on a nonuniform unstructured mesh, primarily composed of hexahedral elements. Attention is given to the mesh refinement, with a maximum grid size set to 0.2 mm to accurately capture the complex flow and complex geometry details within the spinal SAS. Moreover, a time step size of 0.004 s (i.e., duration of cardiac cycle/200) is used to maintain numerical stability and accuracy throughout the transient simulations. Both the grid size and the time step size are selected based on the previous studies [4, 11, 28, 29] and our convergence study in Sec. 2.4.

### Canonical spinal SAS geometries

**Fig. 1:**
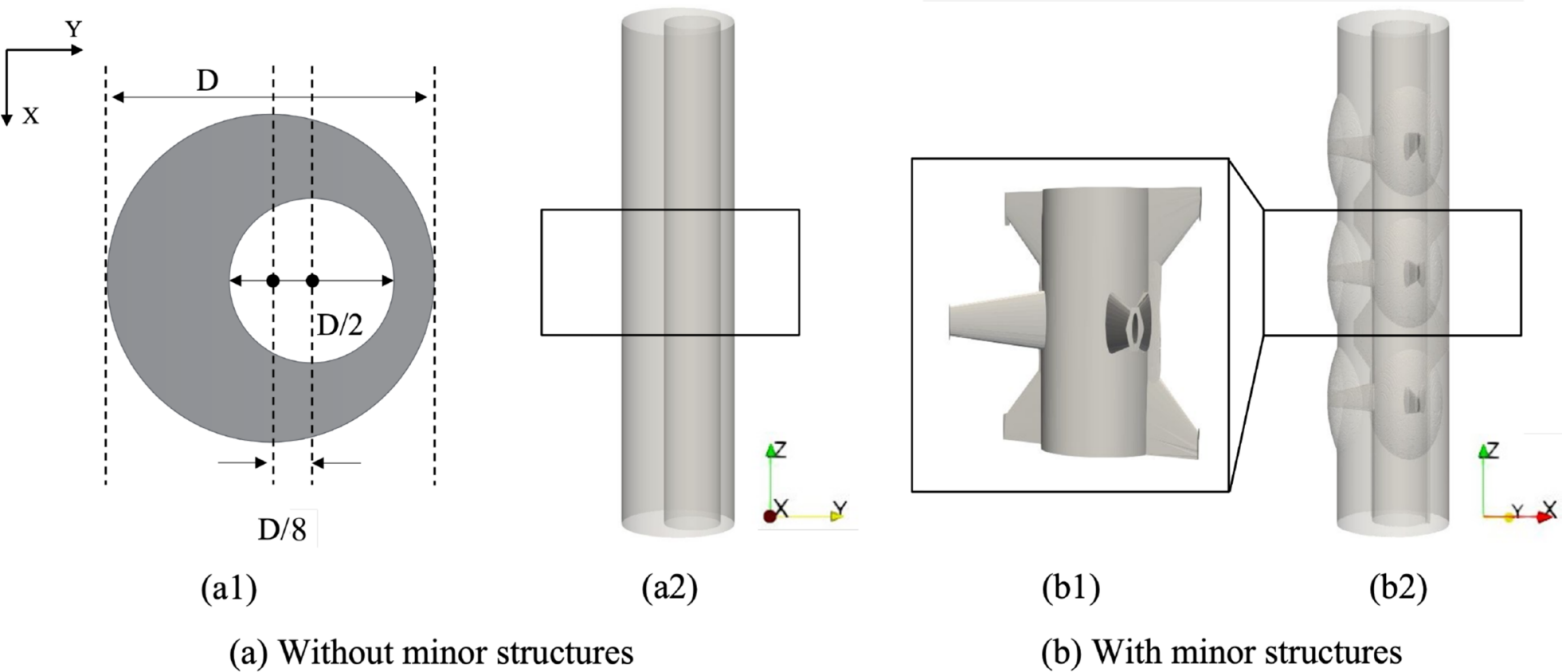
Two canonical spinal SAS geometries investigated in this paper. **a** Views of geometry without minor structures. **a1** Cross section of the simplified geometry (D = 1.8 cm). **a2** The simplified geometry (eccentric annular pipe). **b** Views of geometry with minor structures. **b1** View of the minor structures. **b2** The geometry with minor structures

Two canonical spinal SAS geometries are created for CFD simulations as shown in Fig. 1. One is an eccentric annular pipe with an outer diameter (D) of 1.8 cm, an inner diameter of 9 mm, and an eccentricity (i.e., the ratio of the distance between the two centers to the difference between the inner radius and the outer radius) of 0.5 because a short section of spinal SAS can be regarded as an eccentric annular pipe if the minor features are omitted as many studies [1, 4–7, 21, 30–33] have done. The other geometry is designed based on the eccentric annular pipe by adding simplified minor structures such as nerve roots, denticulate ligaments, and a wavy arachnoid mater. Each of these geometric models encompasses the spinal SAS spanning across three vertebrae, with each vertebra having a height of 2 cm. The dimensions of the geometries are determined based on the information provided in the relevant literature [14, 34–38]. To mitigate the impact of the boundary condition setup on the flow field within the area of interest, an additional buffer zone with a height of 1 cm is appended both above and below the SAS geometries. The data presented and analyzed in this paper are from the boxed SAS section spanning across the middle vertebra.

### CFD simulations

The flow field was computed by solving the incompressible Navier-Stokes equations

1 $$\begin{aligned} & \nabla \cdot {\textbf{u}} = 0, \end{aligned}$$

2 $$\begin{aligned} & \frac{\partial {\textbf{u}}}{\partial t} + ({\textbf{u}} \cdot \nabla ) {\textbf{u}} = -\frac{1}{\rho } \nabla p + \nu \nabla ^2 {\textbf{u}}, \end{aligned}$$

where ${\textbf{u}}$ and *p* describe the velocity and pressure fields, respectively, $\rho$ is the CSF’s density and $\nu$ is the CSF’s kinematic viscosity. In this study, it is assumed that the pulsation of the CSF is solely driven by the heartbeat. The inlet boundary condition is applied at the top, using a profile of CSF flow rate over time. The profile is obtained by adjusting the profile from the literature to ensure a zero temporal mean and a stroke volume of 0.4 mL through the top surface of the box around the middle vertebra (i.e., region of interest) in Fig. 1. The goal of this adjustment is to ensure that the flow field in the region of interest is representative of a typical middle vertebra of the entire human spine, reflecting the average conditions observed across all vertebrae by assuming a linearly decreasing stroke volume along the spine. For simplicity, we will refer to this as the “representative vertebra” throughout the remainder of this paper. A zero Dirichlet boundary condition (i.e., p = 0) is applied for the pressure at the top. A zero-gradient outlet boundary condition for both velocity and pressure (i.e., dw/dz = 0, dp/dz = 0) is used at the bottom. In our study, ’top’ refers to the position at the far end along the positive z-axis, aligned with the caudocranial direction, and ’bottom’ refers to the position at the far end along the negative z-axis, aligned with the craniocaudal direction, as depicted in Fig. 1. The compliance of the arachnoid membrane is assumed to be uniform, allowing for approximately 0.027 mL of CSF to be accommodated by each vertebra during each pulsation. It was found that the displacement of the membrane is comparable to the cell size. Consequently, rather than employing fluid–structure interaction (FSI), a velocity profile derived from the pulsatile flow rate and scaled appropriately is utilized on the outer surfaces of the geometries to emulate the compliance.

Performing a full FSI analysis is computationally expensive and subject to uncertainties in parameter selection and modeling assumptions. We use a velocity profile on the wall to represent the effect of arachnoid displacement. While this approach reduces computational complexity, it serves as an approximation and does not fully replicate the secondary flows resulting from a moving boundary. Although the radial displacement velocity is smaller than the inlet axial velocity, its inclusion is essential due to the slenderness of the geometry. The radial velocity, when scaled by the thin layer between the arachnoid mater and the spinal cord, influences the overall transport dynamics in the axial direction, which is scaled by the height of the geometry.

A zero-gradient boundary condition is applied for pressure (i.e., dp/dn = 0, where n is the direction normal to the surface) on the outer wall. And the surrounding tissues (inner walls and nerve roots) are assumed to be rigid.

The steady streaming velocity field can be determined by calculating the time average of the Eulerian velocity over a cycle once a periodic state has been reached [1, 21]

3 $$\begin{aligned} \langle {\textbf{u}}\rangle _{SS} = \frac{1}{T}\int _{0}^{T} {\textbf{u}}(t) dt = [\langle u \rangle _{SS}, \langle v \rangle _{SS}, \langle w \rangle _{SS}]^T, \end{aligned}$$

where T is the duration of the cardiac cycle. The intensity of steady streaming on each Z-normal cross section along the spinal cord can be quantified by the area integral

4 $$\begin{aligned} Q_{SS}(z=c) = \frac{1}{2}\iint \limits _{z = c} \vert \langle w \rangle _{SS} \vert \, dS. \end{aligned}$$

Here are some dimensionless numbers characterizing the behavior of the drug particles suspended in the CSF flow after the injection jet has been dissipated

5 $$\begin{aligned} & Pe = \frac{L_{st}u_{st}}{D} \sim 10^5>> 1, \end{aligned}$$

6 $$\begin{aligned} & Re_p = \frac{u_{st} d_p}{\nu } \sim 0.25 < 1, \end{aligned}$$

7 $$\begin{aligned} & Stk = \frac{t_{0}u_{st}}{d_p} < 0.01, t_{0} = \frac{\rho _p d^2_p}{18\mu }, \end{aligned}$$

where $L_{st}$, $u_{st}$ are the stroke length and stroke velocity of the CSF flow, $\mu$ is the CSF dynamic viscosity, $d_p$ and $\rho _p$ are the particle’s diameter and density, respectively. Given that the Peclet number, *Pe*, is significantly greater than 1, and the Stokes number, *Stk*, is substantially less than 1, the dispersion of the drug can be accurately modeled using Lagrangian particle tracking

8 $$\begin{aligned} \frac{d{\textbf{x}}_i}{dt} = {\textbf{u}}({\textbf{x}}_i,t), \end{aligned}$$

where ${\textbf{x}}_i$ is the position vector of particle ‘i’ over time. Approximately 1000 particles were uniformly distributed on each one of the 16 uniformly spaced Z-normal cross sections over the representative vertebra. The cycle-averaged Lagrangian velocity at the location where the particle ‘i’ was released at the beginning of a cycle (t = 0) when a periodic state has been reached can be computed as shown below [1, 21]

9 $$\begin{aligned} \langle {\textbf{u}}\rangle _{L}({\textbf{x}}_{i,t=0}) = \frac{1}{T}\int _{0}^{T} \frac{d{\textbf{x}}_i(t)}{dt} \, dt = [\langle u \rangle _{L}, \langle v \rangle _{L}, \langle w \rangle _{L}]^T. \end{aligned}$$

The cycle-averaged Stokes drift velocity at the same location can be computed as [1, 21]

10 $$\begin{aligned} \langle {\textbf{u}}\rangle _{SD}({\textbf{x}}_{i,t=0}) = \langle {\textbf{u}}\rangle _{L}({\textbf{x}}_{i,t=0}) - \langle {\textbf{u}}\rangle _{SS}({\textbf{x}}_{i,t=0}) = [\langle u \rangle _{SD}, \langle v \rangle _{SD}, \langle w \rangle _{SD}]^T. \end{aligned}$$

### Quantification of zones with upward and downward cycle-averaged velocities

The optimization involves a number of key considerations. The process should be designed to minimize the time taken for the administered drugs to reach their targeted regions, thereby ensuring a timely therapeutic response. Furthermore, the quantity of the drug reaching these targeted sites should be maximized to enhance the treatment’s overall effectiveness. The importance, therefore, lies in the quantification of the average speed at which the drugs can be transported upwards and the proportion of drug particles that moves upwards.

The average upward steady streaming velocity on each Z-normal cross section can be quantified by dividing $Q_{SS}$ by the area where upward steady streaming velocity is present

11 $$\begin{aligned} \overline{\langle w \rangle }_{SS, \uparrow } (z=c)= \frac{Q_{SS}(z=c)}{ \iint _{\{ (x, y, z=c): \langle w \rangle _{SS}(x,y)>0 \}} dxdy }. \end{aligned}$$

Similarly, the normalized upward cycle-averaged Stokes drift or Lagrangian velocities can be calculated by dividing the sum of upward cycle-averaged velocities by the number of particles that exhibit upward cycle-averaged velocity, as follows:

12 $$\begin{aligned} & \overline{\langle w \rangle }_{SD, \uparrow } (z=c)= \frac{\sum _{\left\{ i \in {\mathcal {N}}_{z=c} |\langle w \rangle _{SD}({\textbf{x}}_{i,t=0})>0 \right\} } \langle w \rangle _{SD}({\textbf{x}}_{i,t=0})}{\left| \left\{ i \in {\mathcal {N}}_{z=c} |\langle w \rangle _{SD}({\textbf{x}}_{i,t=0})>0 \right\} \right| }, \end{aligned}$$

13 $$\begin{aligned} & \overline{\langle w \rangle }_{L, \uparrow } (z=c)= \frac{\sum _{\left\{ i \in {\mathcal {N}}_{z=c} |\langle w \rangle _{L}({\textbf{x}}_{i,t=0})>0 \right\} } \langle w \rangle _{L}({\textbf{x}}_{i,t=0})}{\left| \left\{ i \in {\mathcal {N}}_{z=c} |\langle w \rangle _{L}({\textbf{x}}_{i,t=0})>0 \right\} \right| }, \end{aligned}$$

where ${\mathcal {N}}_{z=c}$ refers to the set of particles placed on the cross section of ${z=c}$.

The corresponding Strouhal numbers, which can be conceptualized as the number of pulsations required to transport a drug particle from the bottom to the top of the vertebra, are defined as follows:

14 $$\begin{aligned} & St_{SS, \uparrow } = \frac{\textrm{L}^2 f_{cc}}{\int _{0}^{\textrm{L}} \overline{\langle w \rangle }_{SS, \uparrow } dz}, \end{aligned}$$

15 $$\begin{aligned} & St_{SD, \uparrow } = \frac{\textrm{L}^2 f_{cc}}{\int _{0}^{\textrm{L}} \overline{\langle w \rangle }_{SD, \uparrow } dz}, \end{aligned}$$

16 $$\begin{aligned} & St_{L, \uparrow } = \frac{\textrm{L}^2 f_{cc}}{\int _{0}^{\textrm{L}} \overline{\langle w \rangle }_{L, \uparrow } dz}, \end{aligned}$$

where $\textrm{L}$ is the height of vertebra, $f_{cc}$ refers to the cardiac cycle frequency.

Assuming that the drug particles are uniformly suspended in CSF, the proportion of particles transported upward due to steady streaming on each Z-normal cross section can be measured by the ratio of the area where the upward steady streaming velocity occurs to the total area of the cross section:

17 $$\begin{aligned} A_{SS, \uparrow } (z=c)= \frac{ \iint _{\{ (x, y, z=c): \langle w \rangle _{SS}(x,y)>0 \}} dxdy }{\textrm{A}_{z=c}}. \end{aligned}$$

Similarly, the normalized area of the region with upward cycle-averaged Stokes drift or Lagrangian velocities can be computed by dividing the number of particles that exhibit upward cycle-averaged velocity by the total number of particles, as follows:

18 $$\begin{aligned} & A_{SD, \uparrow } (z=c)= \frac{\left| \left\{ i \in {\mathcal {N}}_{z=c}|\langle w \rangle _{SD}({\textbf{x}}_{i,t=0})>0 \right\} \right| }{\left| {\mathcal {N}}_{z=c} \right| }, \end{aligned}$$

19 $$\begin{aligned} & A_{L, \uparrow } (z=c)= \frac{\left| \left\{ i \in {\mathcal {N}}_{z=c}|\langle w \rangle _{L}({\textbf{x}}_{i,t=0})>0 \right\} \right| }{\left| {\mathcal {N}}_{z=c} \right| }. \end{aligned}$$

### Convergence study

**Fig. 2:**
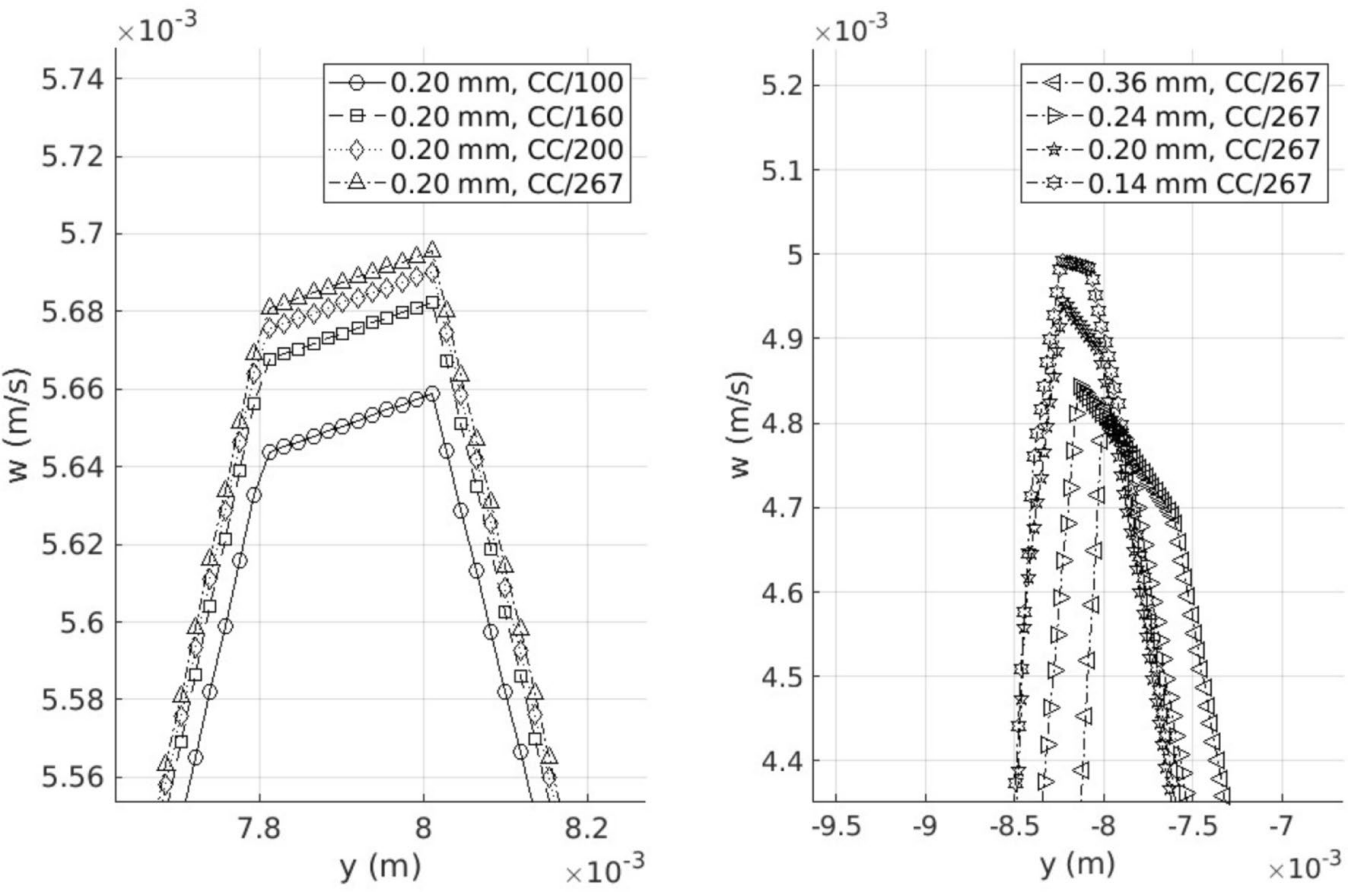
The axial velocity (*w*) along the axis of symmetry of the plane at z = 0.04 m at the first diastolic peak for different time step sizes (CC/100, CC/160, CC/200 and CC/267, where ‘CC’ denotes the length of cardiac cycle) and cell sizes (0.36 mm, 0.24 mm, 0.20 mm and 0.14 mm)

Convergence study has been conducted to help select appropriate mesh size and time step size. Relative errors in the velocity field are quantified for a simulation with a time step size of 1/100, 1/160, 1/200 and 1/267 of the length of the cardiac cycle and with a maximum cell size of 0.36 mm, 0.24 mm, 0.20 mm and 0.14 mm as shown in Table 1. Figure 2 illustrates velocity values obtained from simulations conducted under various setups, providing a comparative visualization of the results. Following the convergence study results, a time step size set to 1/200 of the cardiac cycle duration and a mesh with a maximum cell size of 0.2 mm have been deemed sufficiently fine for our study.

**Table 1:** Relative error for different temporal resolutions (CC/100, CC/160, CC/200 and CC/267, where ‘CC’ denotes the length of cardiac cycle) and spatial resolutions (0.36 mm, 0.24 mm, 0.20 mm and 0.14 mm)

Convergence study	$\Delta$ value	Error (%)
Temporal resolution (0.20 mm)	CC/100 CC/160 CC/200 CC/267	0.35 0.21 0.14 –
Spatial resolution (CC/267)	0.36 mm 0.24 mm 0.20 mm 0.14 mm	6.8 3.3 2.1 –

## Results

### Transient flow fields

**Fig. 3:**
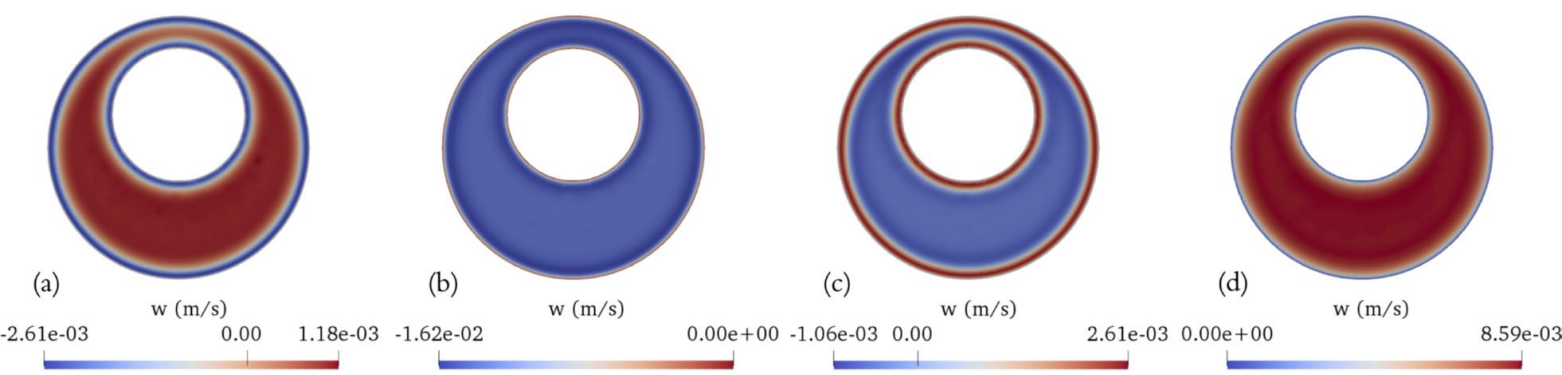
Transient axial velocity (*w*) fields on the middle plane (z = 0.04 m) of the SAS (without minor structures) spanning across the representative vertebra: **a** end of diastole, **b** systolic peak, **c** end of systole, **d** diastolic peak

**Fig. 4:**
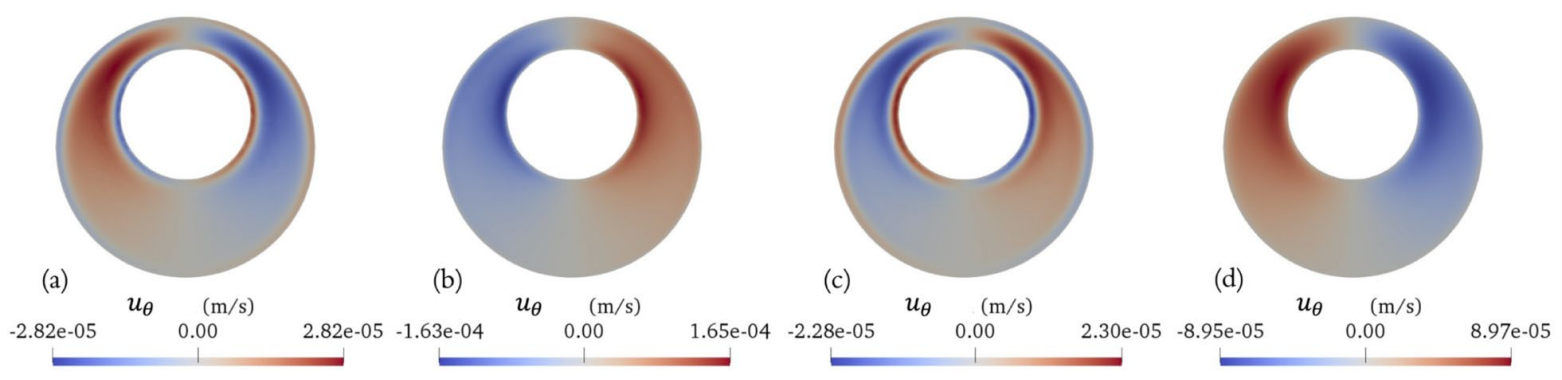
Transient transverse velocity in the azimuthal direction ($u_\theta$) on the middle plane (z = 0.04 m) of the SAS (without minor structures) spanning across the representative vertebra: **a** end of diastole, **b** systolic peak, **c** end of systole, **d** diastolic peak

The transient axial flow (*w*) fields on the middle plane (z = 0.04 m) of the eccentric annular pipe spanning across the representative vertebra at four key time points during the cardiac cycle, which include the end of diastole, the peak of systole, the end of systole, and the peak of diastole are illustrated in Fig. 3. The Womersley number is 9.46 on the widest side of the canal (i.e., $\frac{3D }{16}$ $\sqrt{\frac{2 \pi f_{cc}}{\nu }}$ ) and 3.15 on the narrowest side (i.e., $\frac{D }{16}$ $\sqrt{\frac{2 \pi f_{cc}}{\nu }}$ ). This indicates that the fluid in regions far away from the wall is less susceptible to acceleration. Not surprisingly, it can be observed that the fluid near the wall, where the viscous force is dominant, responds to the changes in pulsation profile more promptly than the fluid situated farther from the wall, where the inertial force takes precedence. For example, when the pulsation transitions from upward to downward flow, the fluid near the wall moves downward while the remainder of the fluid still moves upward, and conversely (i.e., when the pulsation transitions from downward to upward flow), the fluid near the wall moves upward while the rest of the fluid continues to move downward.

The transverse velocity in the azimuthal direction on the middle plane (z = 0.04 m) of the eccentric annular pipe spanning across the representative vertebra at the four key time points during the cardiac cycle are illustrated in Fig. 4. Given the large discrepancy in length scales (i.e., the length of the spinal cord and the perimeter of the spinal cord’s cross section), the azimuthal velocity, which can be deduced from the dimensional analysis of the continuity equation, is nearly two orders of magnitude smaller than the axial velocity. As the pulsation transitions from diastole to systole, the fluid begins to move from the wider side of the canal towards the narrower side due to the relatively high pressure generated on the wider side. Conversely, when the pulsation shifts from systole to diastole, the fluid moves in the opposite direction, shifting back towards the wider side due to the relatively low pressure generated on the wider side. Similar to previous observations, the fluid near the wall, where viscous forces dominate, exhibits a quicker response. The azimuthal velocity magnitude peaks near the inner cylindrical wall, where the fluid experiences the highest viscous shear stress. Figures 3 and 4 present results that are similar to those shown in Reference [21] and are included here to establish a baseline for comparison with the effects of minor structures shown in subsequent figures. These results have been previously studied in Reference [21].

**Fig. 5:**
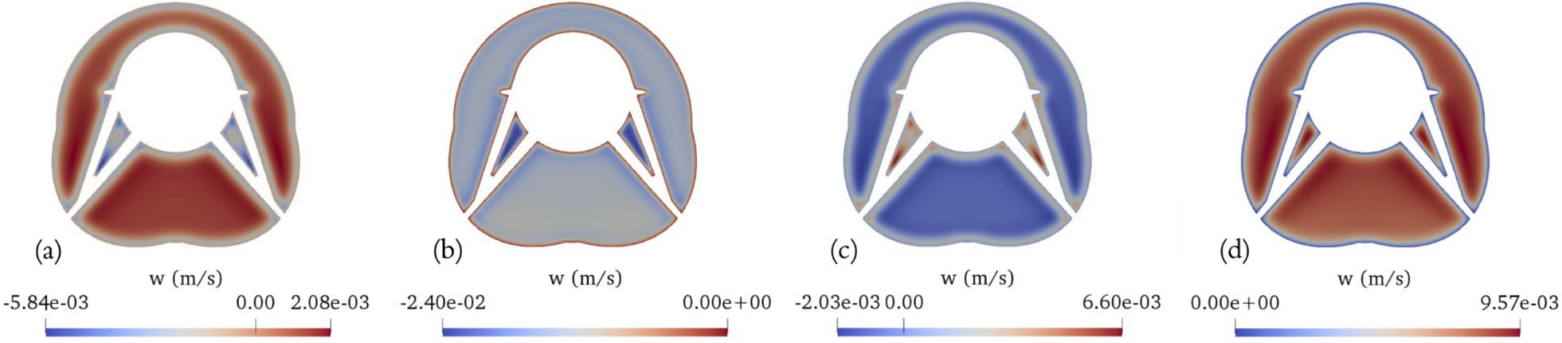
Transient axial velocity (*w*) fields on the middle plane (z = 0.04 m) of the SAS (with minor structures) spanning across the representative vertebra: **a** end of diastole, **b** systolic peak, **c** end of systole, **d** diastolic peak

**Fig. 6:**
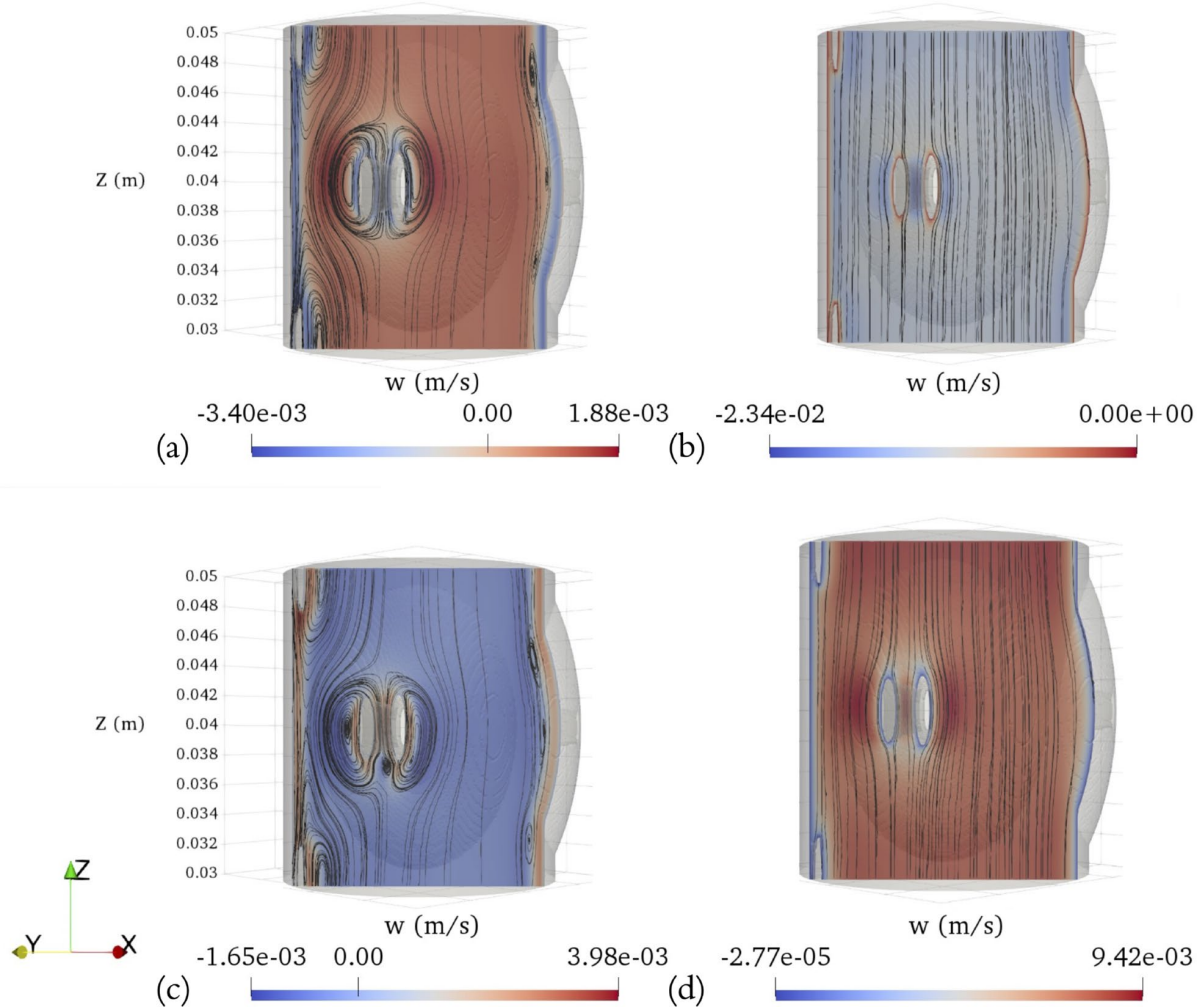
Transient axial velocity (*w*) fields and streamlines on a cross section of the SAS (with minor structures) spanning across the representative vertebra. The cross section has a normal vector of [1,1,0] and intersects the point ( $-$0.003 m, $-$0.003 m, 0 m) with respect to the outer circle’s center: **a** end of diastole, **b** systolic peak, **c** end of systole, **d** diastolic peak

The transient axial flow (*w*) fields on the middle plane (z = 0.04 m) of the geometry with added minor features spanning across the representative vertebra at four key time points during the cardiac cycle, which include the end of diastole, the peak of systole, the end of systole, and the peak of diastole are illustrated in Fig. 5. The complex geometries generate extra areas in the middle of the domain where viscous forces dominate, leading to an increased range in velocity magnitude. High velocity magnitudes are concentrated around narrower regions, especially the areas between the anterior and posterior roots. Larger velocity magnitudes are also observed in the vicinity outside of the nerve roots compared to the areas near the spinal cord and arachnoid surfaces due to a combination of factors: viscosity, transient vortices generated by a pulsatile flow, and the acceleration occurring around the immersed object, which is more pronounced in Fig. 6. It presents the transient axial flow (*w*) fields and streamlines at four crucial time points. These are captured on a cross section of the geometry that includes minor features and spans the representative vertebra. This particular cross section is defined by a normal vector [1,1,0] and intersects the point ($-$0.003 m, $-$0.003 m, 0 m) relative to the center of the outer circle. When one phase concludes, the change in flow direction near the wall induces the formation of vortices near the nerve roots, denticulate ligaments, and wavy outer surface. Notably, a strong vortex is formed around each nerve root, enveloping additional vortex situated along the outer side of each respective nerve root. Additionally, three weak vortices are created around the wavy outer structure. The positioning of these vortices also slightly shifts according to flow transitions: when the flow transitions from upward to downward, vortices appear higher, and they situate slightly lower when the flow transitions from downward to upward. The reason behind this is the existence of a stagnation zone that forms in the wake of the immersed object as the CSF flows over it. This stagnation zone can essentially be considered an expanded virtual volume of the immersed object enclosed by a virtual wall.

### Cycle-averaged velocity fields

**Fig. 7:**
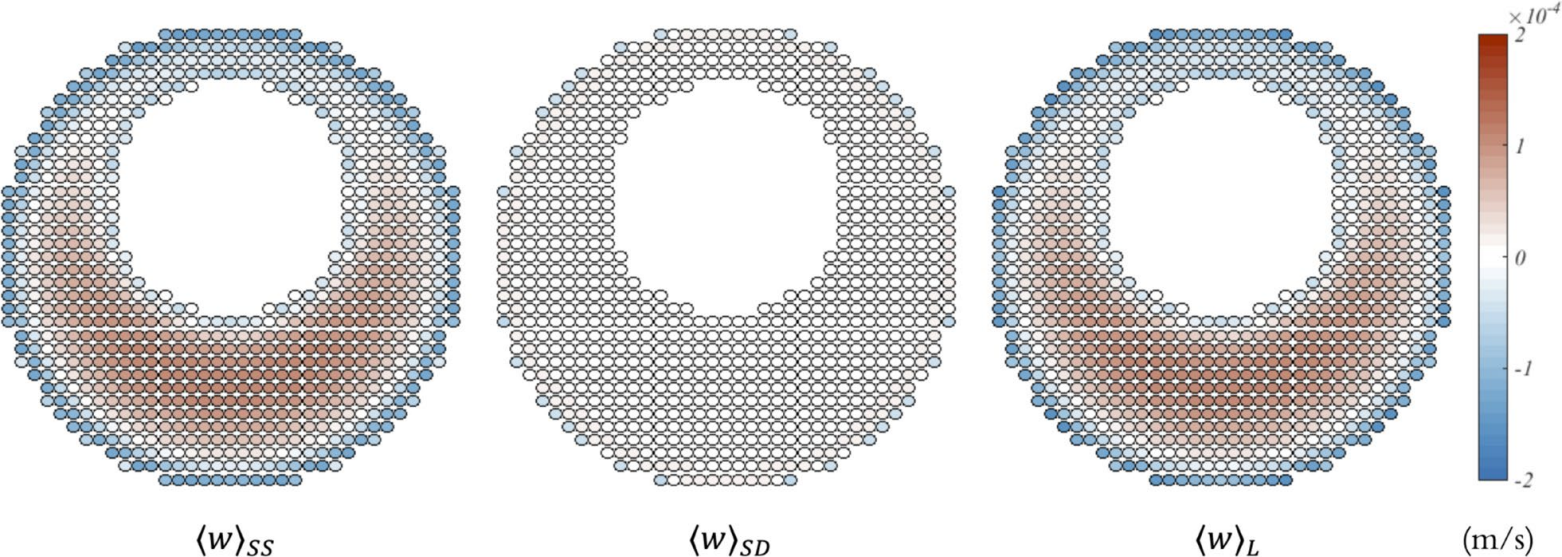
Axial steady streaming ($\langle w \rangle _{SS}$), cycle-averaged Stokes drift ($\langle w \rangle _{SD}$) and cycle-averaged Lagrangian ($\langle w \rangle _{L}$) velocity fields on the middle plane (z = 0.0400 m) of the SAS (without minor structures) spanning across the representative vertebra

The axial steady streaming ($\langle w \rangle _{SS}$), cycle-averaged Stokes drift ($\langle w \rangle _{SD}$), and cycle-averaged Lagrangian ($\langle w \rangle _{L}$) velocity fields are computed on the median plane (z = 0.0400 m) of the simplified SAS, which spans across the representative vertebra without minor structures as depicted in Fig. 7. It is observed that the steady streaming velocity tends to be downward near the wall while it is upward elsewhere, thus forming a downward zone on the narrow side and near the wall. This encloses an upward area in the relatively wider region, which is consistent with the findings reported in the literature [7]. This distribution can be attributed to the transverse convective acceleration, which leads to more fluid, governed by inertia, being transported upward, and conversely, where the viscous force is dominant, the fluid moves downward. The discrepancy in the magnitude of the steady streaming velocity near the inner and outer surfaces may be attributed to an excessively high shear stress near the inner wall, particularly for the flow through the annular pipe. The results illustrate that the axial steady streaming and cycle-averaged Lagrangian velocity fields are nearly identical, primarily because the axial cycle-averaged Stokes drift velocities are an order of magnitude smaller than either of them. Although not illustrated here, the cycle-averaged velocity fields appear to remain almost the same along the Z-axis within the representative vertebra. However, over the entire extent of the spinal SAS, the stroke volume decreases as the z-value reduces due to compliance. As a result, the cycle-averaged velocity fields gradually scale down and eventually disappear at the terminal end of the spinal canal. Figure 7 shows similar findings to those in Reference [1] and is incorporated herein to compare how minor structures affect the results in later figures.

**Fig. 8:**
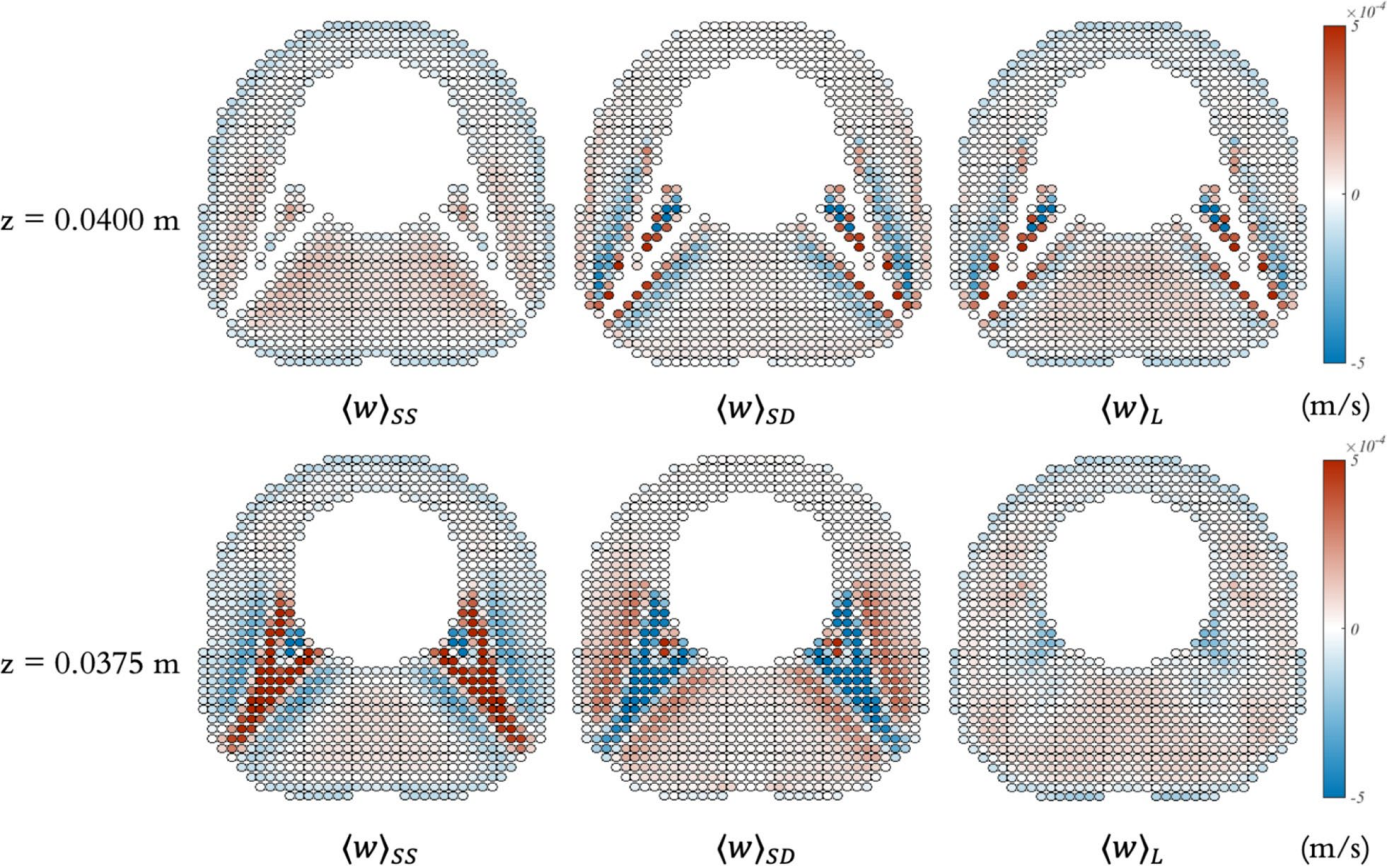
Axial steady streaming ($\langle w \rangle _{SS}$), cycle-averaged Stokes drift ($\langle w \rangle _{SD}$) and cycle-averaged Lagrangian ($\langle w \rangle _{L}$) velocity fields on cross sections (z = 0.0400 m, z = 0.0375 m) of the SAS (with minor structures) spanning across the representative vertebra

**Fig. 9:**
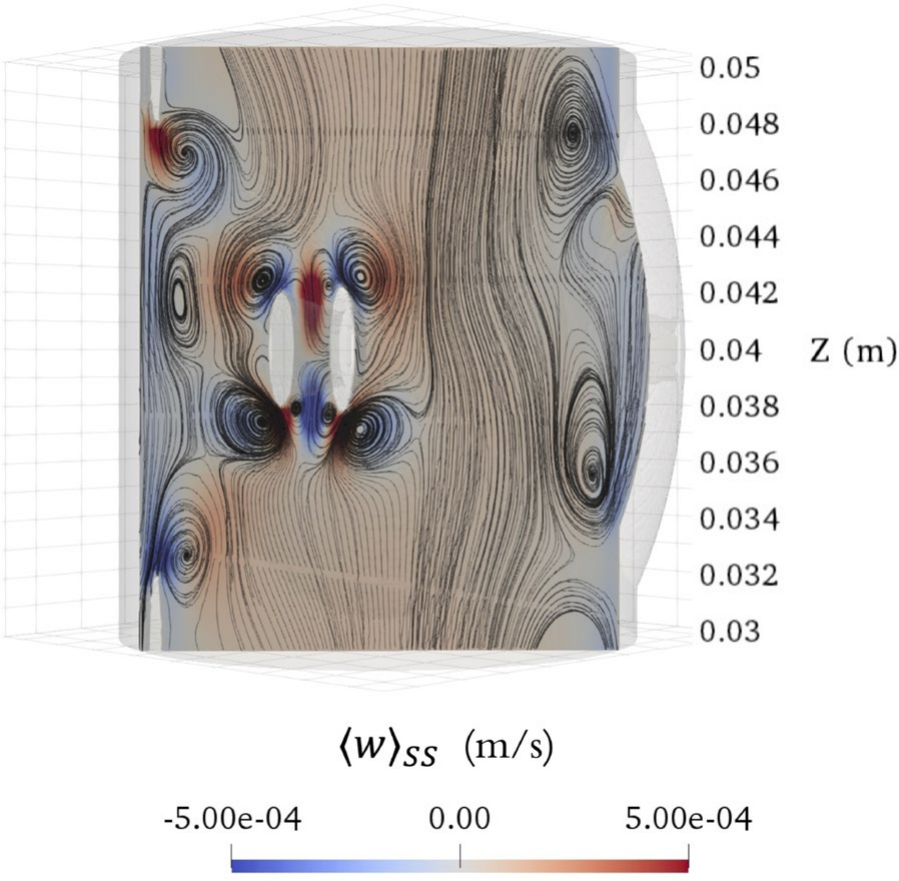
Axial steady streaming velocity field ($\langle w \rangle _{SS}$) on a cross section of the SAS (with minor structures) spanning across the representative vertebra. The cross section has a normal vector of [1,1,0] and intersects the point ( $-$0.003 m, $-$0.003 m, 0 m) with respect to the outer circle’s center

**Fig. 10:**
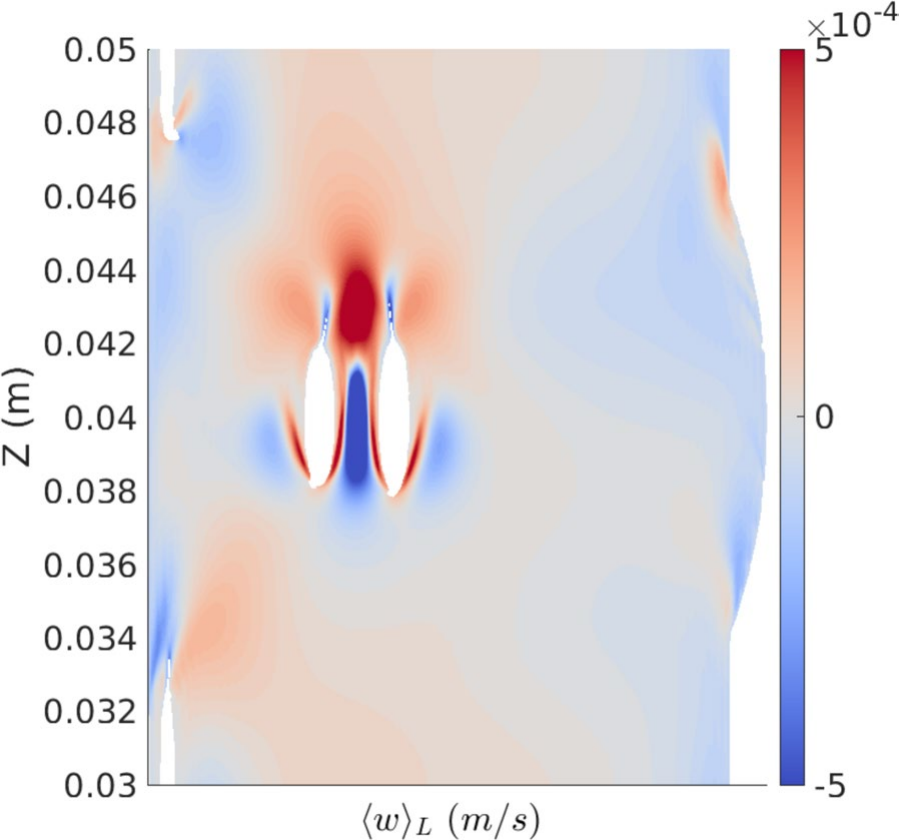
Cycle-averaged Lagrangian ($\langle w \rangle _{L}$) velocity field on a cross-sectional plane of the SAS geometry (with minor structures), spanning across the representative vertebra. The cross section, with a normal vector of [1,1,0], intersects the point ($-$0.003 m, $-$0.003 m, 0 m) relative to the center of the outer circle. The field was obtained by interpolating the particle-based velocities to create a continuous representation, providing a smooth depiction of transport dynamics across the plane.

Figures 8 and 9 show the cycle-averaged velocity fields within the spinal SAS featuring minor structures and spanning across the representative vertebra. Figure 8 presents the axial steady streaming ($\langle w \rangle _{SS}$), cycle-averaged Stokes drift ($\langle w \rangle _{SD}$), and cycle-averaged Lagrangian ($\langle w \rangle _{L}$) velocity fields on two different cross-sections, z = 0.0400 m and z = 0.0375 m, whereas Fig. 9 depicts the axial steady streaming velocity ($\langle w \rangle _{SS}$) on a cross section with an orientation, characterized by a normal vector of [1,1,0] and intersecting the point ( $-$0.003 m, $-$0.003 m, 0 m), referenced to the center of the outer circle. The cycle-averaged velocity fields become significantly complex and exhibit dramatic changes along the Z-axis when minor structures are considered. Steady streaming vortices occur around the minor structures and exhibit an overall mirror symmetry about the middle plane (z = 0.04 m). Notably, strong, steady streaming vortices form above and below the nerve roots and denticulate ligaments due to the small offset in the location of the transient vortices formed when the pulsation phase alters. Concurrently, a broad region on the wider side maintains a consistent upward cycle-averaged Eulerian velocity throughout the vertebra. In addition, observations suggest that the magnitude of cycle-averaged Stokes drift velocities is comparable to that of steady streaming velocities when the minor structures are considered. However, where steady streaming vortices occur, Stokes drift introduces cycle-averaged velocity of opposite direction to the steady streaming velocity, yet with comparable magnitudes. This causes the overall magnitudes of the cycle-averaged Lagrangian velocities to be maintained at relatively low levels.

Figure 10 shows the cycle-averaged Lagrangian velocity field in the z-direction ($\langle w \rangle _{L}$) on this specific cross-sectional area of the spinal SAS geometry with minor structures. The contour highlights distinct regions of enhanced particle transport near the minor structures, as evidenced by the alternating high (red) and low (blue) velocity zones. These structures induce stronger velocity gradients and flow recirculation compared to geometries without minor structures, thereby promoting increased mixing and transport efficiency. The presence of localized high-velocity regions (red) adjacent to the structures suggests that these minor features play a key role in disrupting flow patterns and enhancing overall particle distribution within the system.

To compute this field, particles were generated within the cross-sectional area, and their displacement in the z-direction was observed over one cycle. The cycle-averaged field was calculated by subtracting the final from the initial positions of the particles, then dividing by the cycle duration. A continuous field was obtained by interpolating the values between particles. Due to boundary conditions, some particles were missed, leading to the appearance of very small circular gaps on top of the semi-elliptical shapes.

### Quantitative analysis of upward and downward cycle-averaged velocity zones

**Fig. 11:**
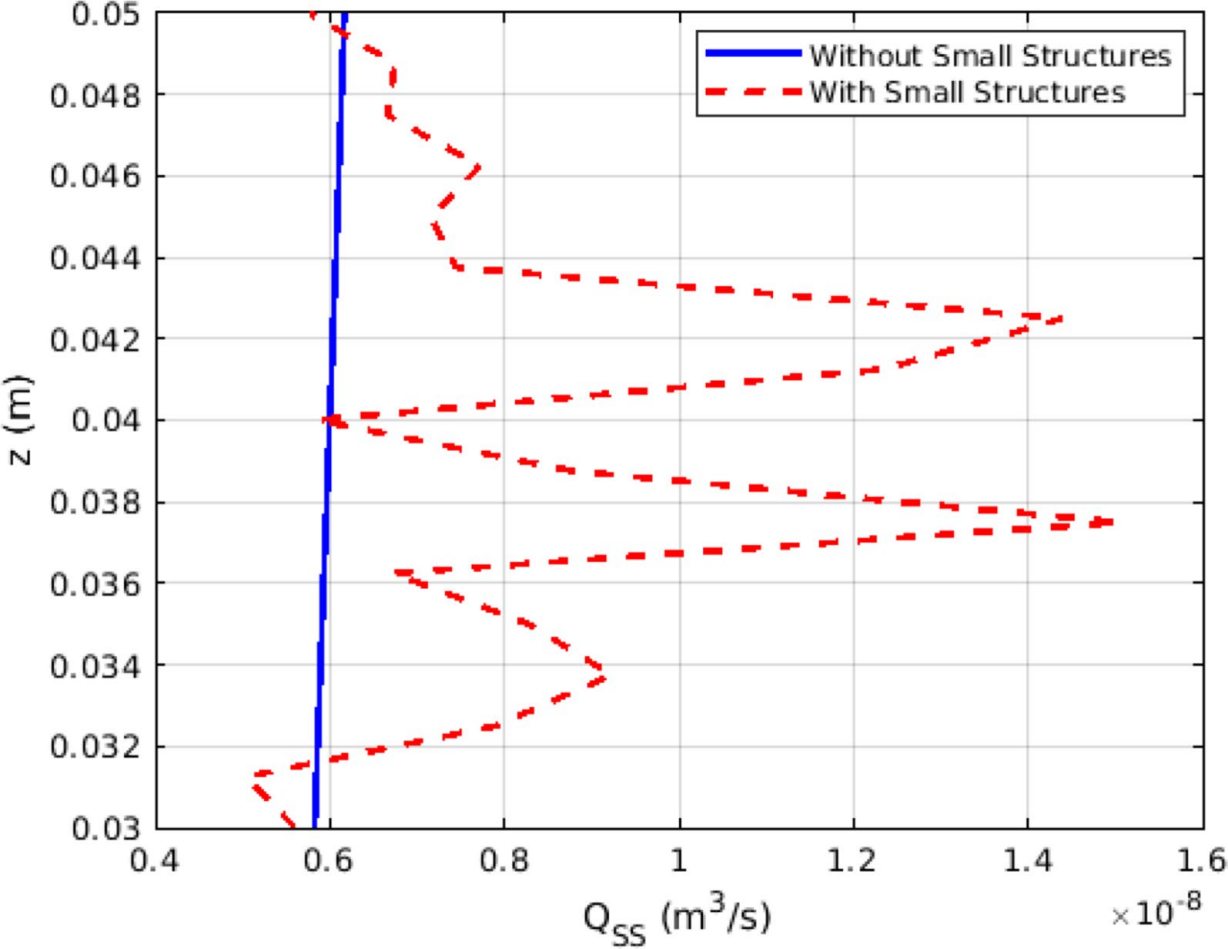
The plots of the intensity of steady streaming ($Q_{SS}$) within the SAS geometries spanning across the representative vertebra

**Fig. 12:**
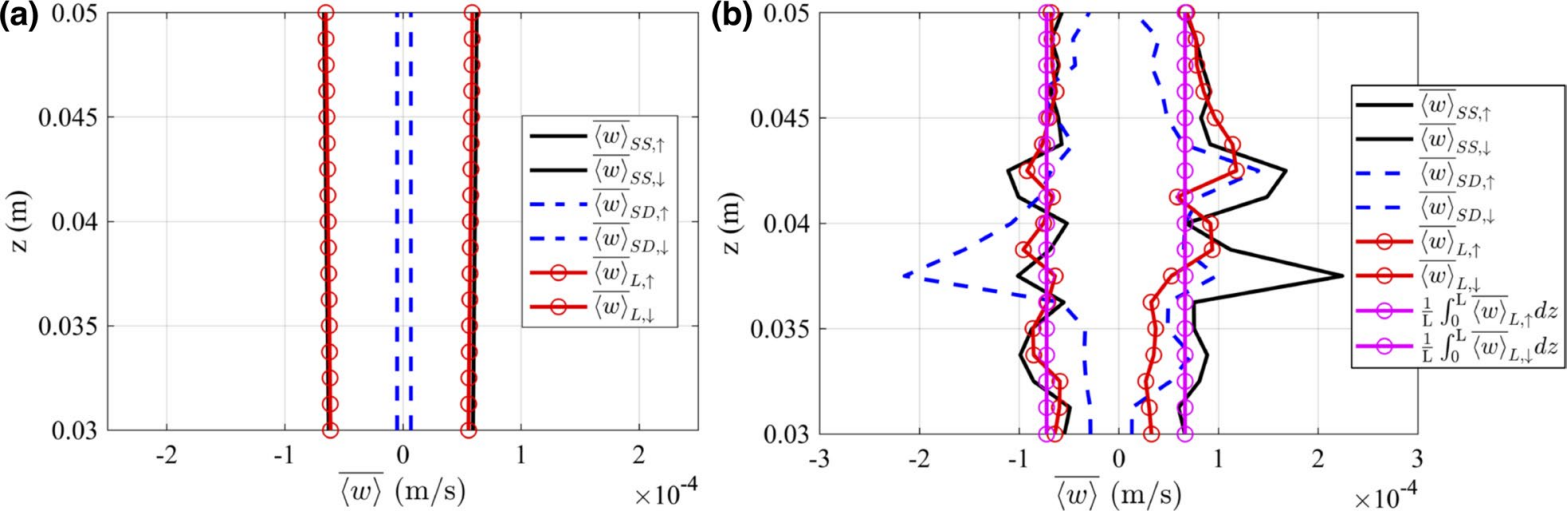
The plots of both the average upward and downward steady streaming velocity ($\overline{\langle w \rangle }_{SS, \uparrow }$, $\overline{\langle w \rangle }_{SS, \downarrow }$), normalized upward and downward cycle-averaged Stokes drift velocities ($\overline{\langle w \rangle }_{SD, \uparrow }$, $\overline{\langle w \rangle }_{SD, \downarrow }$) and normalized cycle-averaged Lagrangian velocities of particles moving upward and downward ($\overline{\langle w \rangle }_{L, \uparrow }$, $\overline{\langle w \rangle }_{L, \downarrow }$) within the SAS geometries spanning across the representative vertebra: (a) without minor structures, (b) with minor structures

Figure 11 displays the plots for the intensity of steady streaming ($Q_{SS}$) within the SAS geometries that span across the representative vertebra. The presence of minor structures, especially the nerve roots, can be observed to enhance the steady streaming by up to 2.5 times. The intensity of steady streaming indicates the dispersion of the cycle-averaged Eulerian velocity field, which implies that a high intensity of steady streaming signifies an increase in both the upward and downward cycle-averaged Eulerian velocity. Therefore, as depicted in Fig. 12, we observe an enhancement in the average steady streaming velocity, particularly in the vicinity of the nerve roots.

Figure 12 presents the average upward and downward steady streaming velocity ($\overline{\langle w \rangle }_{SS, \uparrow }$, $\overline{\langle w \rangle }_{SS, \downarrow }$), normalized upward and downward cycle-averaged Stokes drift velocities ($\overline{\langle w \rangle }_{SD, \uparrow }$, $\overline{\langle w \rangle }_{SD, \downarrow }$) and normalized cycle-averaged Lagrangian velocities of particles moving upward and downward ($\overline{\langle w \rangle }_{L, \uparrow }$, $\overline{\langle w \rangle }_{L, \downarrow }$) within SAS geometries spanning the representative vertebra. Figure 12a illustrates the scenario without minor structures, and Fig. 12b shows the effect of the minor features. In the absence of minor structures, the magnitudes of average steady streaming and normalized cycle-averaged Lagrangian velocities appear to be nearly identical, averaging around 0.0571 mm/s over the vertebra for upward direction. This similarity arises due to the fact that the magnitudes of normalized cycle-averaged Stokes drift velocities are relatively small. When minor structures are incorporated into the geometry, there’s an increase in the magnitudes of the average upward and downward steady streaming velocities, as mentioned above. Notably, due to the presence of nerve roots, the average upward steady streaming velocity can be amplified up to 3.7 times. The normalized downward cycle-averaged Stokes drift velocity also escalates up to 3.7 times, similar to the average upward steady streaming velocity but in an opposite direction. Because of the counteracting influences of steady streaming and Stokes drift, the normalized cycle-averaged Lagrangian velocity is relatively suppressed, averaging around 0.0664 mm/s over the vertebra for the upward direction.

**Table 2:** The Strouhal numbers for the three different cycle-averaged velocity fields in the two different vertebra’s geometries

Cases	$St_{SS, \uparrow }$	$St_{SD, \uparrow }$	$St_{L, \uparrow }$
Without minor structures	411	3724	438
With minor structures	263	450	376

The Strouhal numbers for both the simple eccentric annular pipe and the geometry incorporating the minor structures are detailed in Table 2. The Strouhal number essentially represents the number of cardiac cycles needed to transport a particle from the base to the top of a vertebra, assuming it is located in a zone capable of facilitating upward transport. Based on the data presented in the table, it’s evident that the presence of minor structures can enhance the transport rate in all three velocity fields, with the effect being particularly prominent for Stokes drift. When factoring in the minor structures, the upward cycle-averaged Eulerian and Lagrangian velocities experienced an increase of around 56.3 % and 16.5 % on average within the representative vertebra, respectively. Regardless of whether minor structures are considered, it is noted that the Strouhal numbers for the cycle-averaged Lagrangian velocity field always exceed that of the cycle-averaged Eulerian velocity field, especially prominent when the minor structures are considered.

**Fig. 13:**
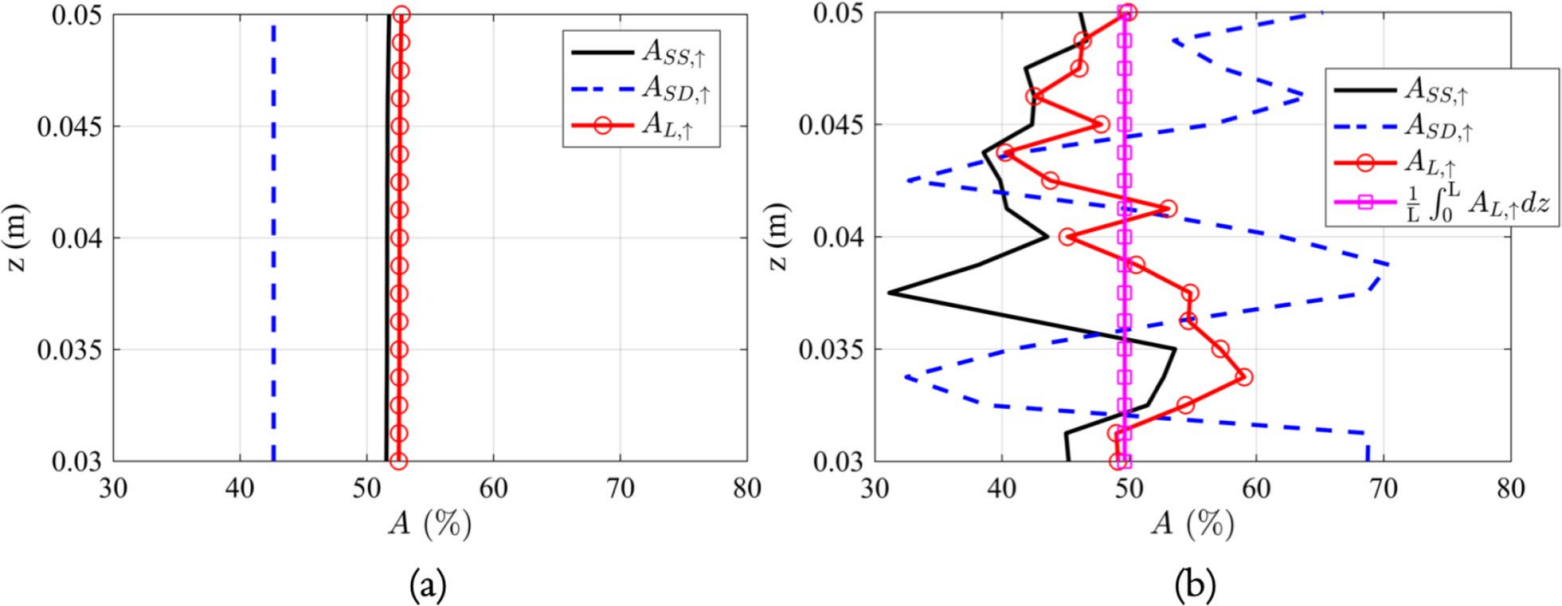
The plots of the normalized area of the region with upward steady streaming velocity ($A_{SS, \uparrow }$), upward cycle-averaged Stokes drift velocity ($A_{SD, \uparrow }$), and upward cycle-averaged Lagrangian velocity ($A_{L, \uparrow }$) within the SAS geometries spanning across the representative vertebra: **a** without minor structures, **b** with minor structures

Figure 13 presents the normalized area of the region with upward steady streaming velocity ($A_{SS, \uparrow }$), upward cycle-averaged Stokes drift velocity ($A_{SD, \uparrow }$), and upward cycle-averaged Lagrangian velocity ($A_{L, \uparrow }$) within the two geometries spanning across the representative vertebra. Figure 13a illustrates the scenario for the SAS geometry without minor structures, while Fig. 13b shows the case when the geometry includes minor structures. The normalized area of the region with upward steady streaming velocity and the normalized area of the region with upward cycle-averaged Lagrangian velocity are nearly identical, averaging about 52% if minor structures are omitted. Upon the inclusion of these minor features, the overall normalized area of the region with upward steady streaming velocity drops to around 44%. The normalized area of the region with upward cycle-averaged Stokes drift velocity varies drastically over the spinal cord. Due to the adjustments induced by Stokes drift, there is an increase in the normalized area of the region with upward cycle-averaged Lagrangian velocity on each Z-normal cross section, leading to an average of approximately 50% over the entire vertebra.

**Fig. 14:**
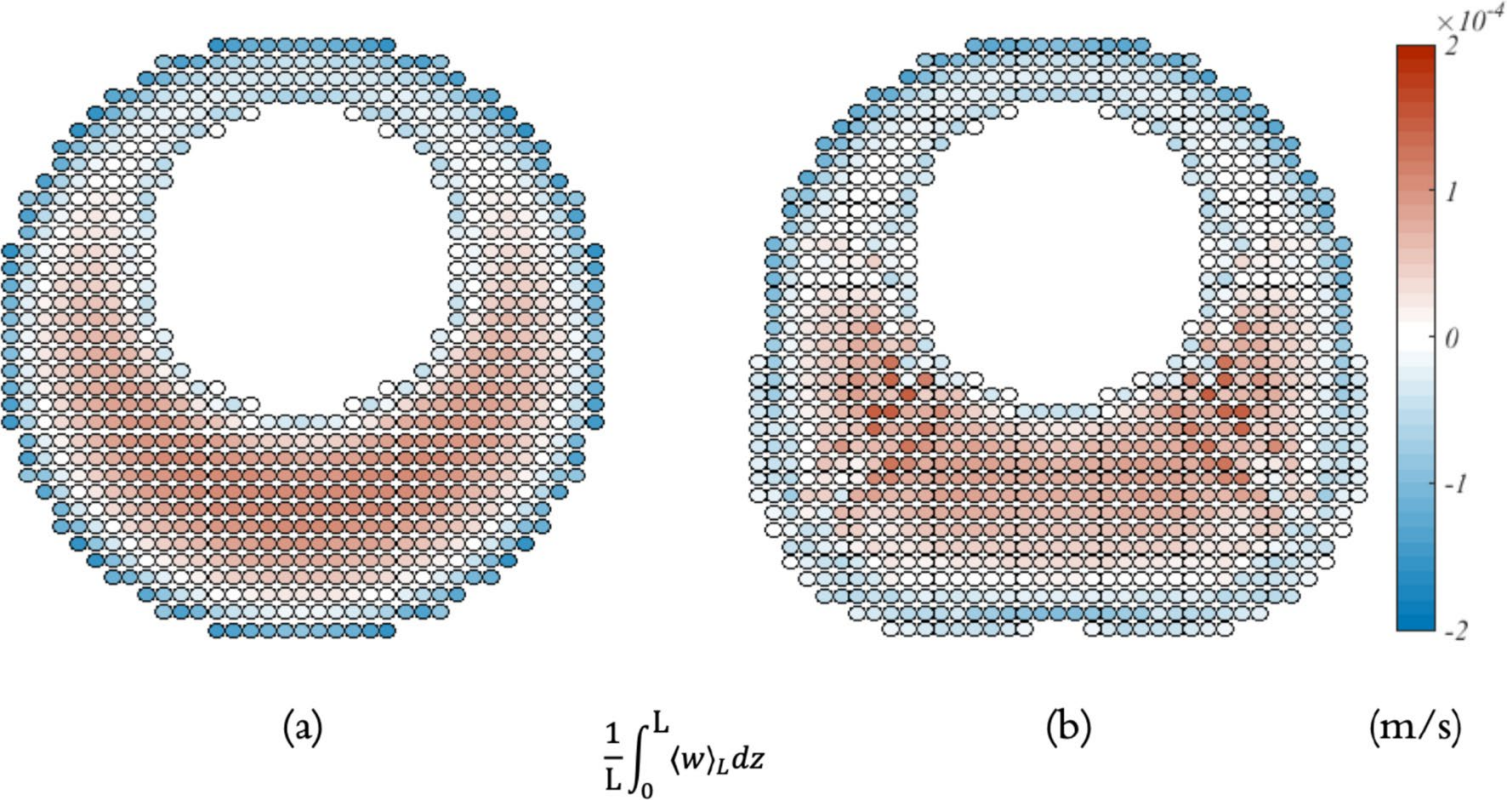
The average of cycle-averaged Lagrangian velocity fields over the Z-axis within the SAS geometries spanning across the representative vertebra: **a** without minor structures, **b** with minor structures

To better understand the mass transport within the spinal SAS, the average of cycle-averaged Lagrangian velocity fields over the Z-axis can be calculated, as depicted in Fig. 14. This calculation assumes a zero velocity outside of the SAS. The observation reveals that the upward transport is concentrated on the wider side, consistent with the findings from the simple eccentric annular pipe. Even though the opposing direction of the steady streaming and Stokes drift can lead to a certain degree of cancellation, noticeable high upward transport can be seen around the nerve roots. Moreover, areas dominated by viscous force, especially near the wall and throughout the entire narrow side, display a downward transport.

## Discussion

Minor structures, essentially including the nerve roots, denticulate ligaments, and the wavy arachnoid membrane, play a critical role in modulating the flow and transport dynamics in the spinal SAS. By including the minor structures, the transient flow fields are different from those of the simple eccentric annular pipe. It is found that the primary vortex surrounding the nerve roots, as well as those secondary vortex adhering to the side face of the nerve roots, which are encircled by the primary vortex, are formed during the pulsation. The steady streaming is intensified by minor structures, particularly around the nerve roots. This amplification can lead to an increase in the magnitudes of both the upward and downward cycle-averaged Eulerian velocities. These findings can be seen in other studies [28, 29] as well. the magnitudes of both the upward and downward cycle-averaged Stokes drift velocities also increase significantly around the nerve roots. Interestingly, this increase tends to be in the opposite direction to the steady streaming velocity. Even though Particle tracking is rarely employed in computational studies of CSF flow within the spinal SAS, this opposite effect has been reported in both theoretical and experimental studies of oscillating flow near wavy boundaries, as outlined in [25]. The inclusion of minor structures can increase the normalized cycle-averaged Lagrangian velocity of particles moving upward by an average of 14% over the entire vertebra, indicating that these structures have the potential to slightly enhance mass transport. In addition, the average of cycle-averaged Lagrangian velocity fields over the Z-axis has a maximum upward velocity of 0.11 mm/s occurring on the sagittal plane in the simple eccentric annular pipe. When the minor features are included, the maximum upward velocity is increased to 0.14 mm/s, occurring in the vertical columns between the nerve roots as shown in Fig. 14. This value aligns well with experimental observations of IT tracer injections [12, 39]. To summarize, minor structures within the spinal SAS influence on the resulting flow fields, creating patterns that deviate from those observed in simplified models, such as an annular pipe. This, in turn, affects the transport dynamics within the system. It is also worth noting that nerve roots could affect the effectiveness of drug delivery. To optimize the injection protocols, it is crucial to prevent the undesirable accumulation of high concentrations of the drug in non-target regions. Such inadvertent trapping of the drug can result in unwanted side effects and compromise the intended therapy by reducing the amount of drug reaching the desired location. Our observations reveal that the volume where fluid movement is nearly stagnant remains almost the same, irrespective of the presence or absence of minor structures (although the volume is not explicitly presented in the results section, it has been calculated for analysis). However, the vortices induced by these minor structures are notable in all three cycle-averaged velocity fields and can trap particles. Thus, it is imperative to include these minor structures, as they are key to a comprehensive grasp of transport dynamics in the spinal SAS by emulating the physiological conditions more accurately. It is important to note that our boundary condition approach, where a velocity profile is imposed on a fixed boundary, serves as an approximation for simulating arachnoid displacement. While this approach captures key aspects of transport dynamics, it does not replicate the secondary flows arising from moving boundaries. This simplification could influence the accuracy of steady streaming predictions, as additional flow components induced by boundary movement are not represented.

Despite the transient vorticity-induced peaks in the Eulerian cycle-averaged velocity field, we generally observed an upward steady streaming zone in the wide region of the geometry, contrasted by a downward steady streaming zone in the narrow region and areas close to the wall. This finding aligns with what has been reported in the literature [7]. The steady streaming velocity field over the third cardiac cycle, detailed in another study [28], was also noted in our investigation, where two upward peaks were observed on the narrow side. Although the difference between the second and third cycles is small, we found a change of the upward peak from the narrow side to the wide side during the first 13 cardiac cycles when a zero initial condition is applied. Since no further shifts were observed over the following 5 cycles, it can be regarded as reaching a periodic state after the first 13 cycles, and any effect from the initial condition has been dissipated. Therefore, the results presented and analyzed in our study are obtained from the 14th cardiac cycle. Some research studies have reduced the computational time by employing the steady streaming velocity field—a cycle-averaged Eulerian mean velocity field that has a velocity value two orders of magnitude smaller than that of the transient flow field. This approach allows for a larger time step in solving transport equations. [11]. This practice arises from the high computational demands frequently encountered in this field of study. Therefore, understanding and quantifying the discrepancy between the Eulerian velocity field and the Lagrangian velocity field, which is more representative of mass transport, becomes crucial for assessing the accuracy of conclusions drawn from studies based on the steady cycle-averaged Eulerian velocity field. In our investigation, we discovered that the Stokes drift plays a significant role when minor structures are present, with its magnitude comparable to that of steady streaming. First, we found that the magnitude of the normalized cycle-averaged Lagrangian velocity of particles moving upward is typically lower than that of the average upward steady streaming velocity. This occurs due to the cancellation effect of peaks induced by the transient vortices in steady streaming and the cycle-averaged Stokes drift velocity fields. Second, the normalized area of the region with upward cycle-averaged Lagrangian velocity is higher than the normalized area of the region with upward steady streaming velocity on each Z-normal cross section, especially when the minor structures are included. These insights demonstrate that merely employing the cycle-averaged Eulerian velocity field to study drug transport may lead to unrealistic representations of the flow fields. Consequently, relying solely on this approach can result in incorrect conclusions.

The interplay between the spinal canal’s geometry and the cycle-averaged Lagrangian velocity field is both complex and significant. In our analysis, we observed a tendency for an upward-directed cycle-averaged Lagrangian velocity zone in the wide region of the geometry, while the downward-directed zones occur on the narrow side and near the outer wall. Interestingly, the Lagrangian velocity fields within the spinal SAS have remained unexplored in previous literature. Nevertheless, support for this relationship can be derived from certain parametric studies designed to investigate IT injection protocols. Research has shown that drug transport occurs more rapidly upward when the drug is injected into a wider region of the spinal SAS, in contrast to injections into narrower regions [8, 9]. Furthermore, the azimuthal angle of the drug injection is important. When the jet is directed not at the spinal cord but at the arachnoid membrane with a nonzero azimuthal angle, it gets confined by both structures in the region where it reaches the wall. It has been found that selecting an azimuthal angle directing the drug into a wider region results in a more rapid upward dispersion compared to an angle that guides the drug into a narrower region [8, 9]. This understanding leads us to a potentially universal principle for optimizing IT injections. Specifically, if the therapeutic goal requires rapid and extensive rostral transport of the drug, injection protocols should be carefully designed to ensure that the majority of the drug enters the wide region where the cycle-averaged Lagrangian velocity is directed upward. Such a tailored approach to injection would leverage the natural flow physics within the spinal canal, potentially enhancing the efficiency and effectiveness of the drug transported in the desired direction. This insight not only adds to our comprehension of the complex interplay between spinal canal geometry and fluid transport but also paves the way for more efficient and effective therapeutic interventions within the spinal SAS.

One of the primary limitations of our study is the need for further validation of the proposed principle using patient-specific geometries and protocols customized based on findings. While our findings suggest a promising correlation between cycle-averaged Lagrangian velocity and simplified spinal SAS geometry, applying the principle to a wide range of individual patient geometries remains untested. We used a simplified geometric model for our simulations, which may not capture the full complexity of geometries derived from medical imaging. The curvature of the spinal cord is neglected in this study. We treated the spinal cord as a rigid structure. Future studies could explore the implications of a deformable spinal cord to better understand the interactions between spinal cord movements and surrounding fluid dynamics. The deformability of tissues is also neglected in our study. While we modeled the compliance of the arachnoid membrane by applying a pulsatile flow velocity profile at the outer membrane, this may not accurately reflect the real dynamics of fluid interacting with flexible walls. Future studies should consider implementing Fluid–Structure Interaction (FSI) models, which may also affect drug dispersion. In addition, we did not include the effects of gravitational force on fluid flow and drug movement. This exclusion may limit the applicability of our findings in situations where gravity significantly influences CSF and drug particle dynamics, especially when the drug’s density varies substantially from that of CSF. Future research should incorporate gravitational effects for a more comprehensive analysis in different settings. Furthermore, our study assumes that CSF flow repeats with the cardiac cycle, based on the idea that CSF flow is primarily driven by cardiac pulsation. However, there is evidence that respiration also influences CSF flow, potentially more than cardiac pulsation [40–42]. Our computational model did not consider the potential impact of respiration on CSF dynamics and drug dispersion. Since respiration can affect intrathoracic and intra-abdominal pressures, and thereby CSF flow, ignoring this physiological aspect could reduce the accuracy of our models. It has also been observed that CSF flow patterns change during sleep [43], which has not been considered in our study. Future studies should aim to incorporate various factors, including respiration and sleep, that can influence CSF flow. It is also worth noting that we assumed that there is no production or absorption of CSF within the spinal canal, leading to a cycle-averaged flow rate through the spinal canal to be zero. However, our current understanding and assumptions might be subject to reevaluation, as the study of CSF remains an active and rapidly evolving area of research. For example, there is an ongoing debate about the net flow of intracranial CSF through the cerebral aqueduct [44–46].

## Conclusion

Our study underscores the crucial role minor structures such as nerve roots, denticulate ligaments, and the wavy arachnoid membrane play in the modulation of flow and transport dynamics within the spinal SAS. The presence of minor structures was shown to increase the normalized cycle-averaged Lagrangian velocity of particles moving upward by an average of 14% over the entire vertebra. Moreover, the inclusion of minor structures produced an increase in all three cycle-averaged velocity fields. We also emphasized the necessity of employing particle tracking in computational studies of mass transport within the spinal SAS. Importantly, we discovered that minor structures exert a significant influence on the cycle-averaged Stokes drift velocity field, leading to a pronounced discrepancy between the cycle-averaged Eulerian and Lagrangian velocity fields. Lastly, the study sheds light on the complex interplay between the spinal canal’s geometry and transport dynamics, underscoring the possibility of optimizing IT injection protocols by harnessing natural flow dynamics within the spinal canal. Our analysis of the flow fields showed a clear pattern: a broad cycle-averaged Lagrangian velocity zone in the wider region of the geometry, contrasted by a downward zone in the narrower region and areas near the wall. By designing injection protocols that direct the majority of the drug into the wide region where the cycle-averaged Lagrangian velocity is directed upward, we can potentially enhance the efficiency and effectiveness of drug transport in the desired direction.

## Data Availability

The raw data are available from the corresponding author on reasonable request.
